# Automated recognition of asymmetric gait and fatigue gait using ground reaction force data

**DOI:** 10.3389/fphys.2023.1159668

**Published:** 2023-03-07

**Authors:** Zixiang Gao, Yining Zhu, Yufei Fang, Gusztáv Fekete, András Kovács, Julien S. Baker, Minjun Liang, Yaodong Gu

**Affiliations:** ^1^ Research Academy of Medicine Combining Sports, Ningbo No. 2 Hospital, Ningbo, China; ^2^ Faculty of Engineering, University of Pannonia, Veszprém, Hungary; ^3^ Savaria Institute of Technology, Eötvös Loránd University, Szombathely, Hungary; ^4^ Faculty of Sports Science, Ningbo University, Ningbo, China; ^5^ Department of Sport and Physical Education, Hong Kong Baptist University, Kowloon, Hong Kong SAR, China; ^6^ Department of Physical and Health Education, Udon Thani Rajabhat University, Udon Thani, Thailand

**Keywords:** symmetry function, fatigue gait, statistical parameter mapping, running, support vector machine

## Abstract

**Introduction:** The purpose of this study was to evaluate the effect of running-induced fatigue on the characteristic asymmetry of running gait and to identify non-linear differences in bilateral lower limbs and fatigued gait by building a machine learning model.

**Methods:** Data on bilateral lower limb three-dimensional ground reaction forces were collected from 14 male amateur runners before and after a running-induced fatigue experiment. The symmetry function (SF) was used to assess the degree of symmetry of running gait. Statistical parameter mapping (Paired sample *T*-test) algorithm was used to examine bilateral lower limb differences and asymmetry changes pre- and post-fatigue of time series data. The support vector ma-chine (SVM) algorithm was used to recognize the gait characteristics of both lower limbs before and after fatigue and to build the optimal algorithm model by setting different kernel functions.

**Results:** The results showed that the ground reaction forces were asymmetrical (SF > 0.5) both pre-and post-fatigue and mainly concentrated in the medial-lateral direction. The asymmetry of the medial-lateral direction increased significantly after fatigue (*p* < 0.05). In addition, we concluded that the polynomial kernel function could make the SVM model the most accurate in classifying left and right gait features (accuracy of 85.3%, 82.4%, and 82.4% in medial-lateral, anterior-posterior and vertical directions, respectively). Gaussian radial basis kernel function was the optimal kernel function of the SVM algorithm model for fatigue gait recognition in the medial-lateral and vertical directions (accuracy of 54.2% and 62.5%, respectively). Moreover, polynomial was the optimal kernel function of the anterior-posterior di-rection (accuracy = 54.2%).

**Discussion:** We proved in this study that the SVM algorithm model depicted good performance in identifying asymmetric and fatigue gaits. These findings can provide implications for running injury prevention, movement monitoring, and gait assessment.

## 1 Introduction

Running biomechanics has received widespread attention from runners, coaches and researchers for the past 30 years (Furlong and Egginton, 2018; Gao, 2022; Xu et al., 2022). Typically, the mean value of bilateral variables (Winter, 1984; Grabowski and Kram, 2008) or the default complete symmetry of both limbs (Kyröläinen et al., 2005; Gao et al., 2020c) was widely used in running biomechanical research. Bilateral limb asymmetry is not considered in these studies since complete symmetry of gait is assumed. Although these methods mentioned above can describe the motion well, it also ignores the false claims and misleading interpretations caused by the asymmetry of bilateral variables (Beck et al., 2018). Neuromuscular asymmetry is a widespread phenomenon occurred in functional tasks (Radzak et al., 2017). Few previous studies have considered that the biomechanical asymmetry of running gait (Beck et al., 2018; Gao et al., 2020b; Mastalerz, 2021), even though this phenomenon is common among healthy people (Furlong and Egginton, 2018; Gao et al., 2020c). Quantitative gait characteristics (e.g., parameters of time and parameters of space) and qualitative gait characteristics (e.g., gait variability and gait asymmetry) are related to running-related injuries, especially among amateur runners (Nakayama et al., 2010; García-Pinillos et al., 2020). Similarly, the effects of gait asymmetry are also an important consideration for motor performance (Simoni et al., 2021). Previous studies have shown that a 10% increase in asymmetry of vertical ground reaction force (GRF) leads to a 3.5% increase in net metabolic power during running (Beck et al., 2018). Another finding was that increased foot contact time asymmetry was associated with increased metabolic costs of running (Beck et al., 2018). Zifchock et al. (2006) found that the asymmetry was 49.8% and 37.5% at the running speed of 3.65 m/s by evaluating the peak lateral and medial GRF of bilateral lower limbs in the running process of healthy individuals, similarly by Williams et al. (1987) reported that 13.8% and 20.2% asymmetry of peak lateral and medial GRF at the speed of 5.36 m/s, suggesting that greater symmetry is associated with faster running speeds. The same conclusion regarding walking gait was reported by previous study (Goble et al., 2003).

Bilateral limb asymmetry may not be evident during the initial stages of running but may arise as muscle fatigue and/or exercise intensity changes (Arampatzis et al., 1999; Xiang et al., 2022a). Mizrahi et al. (2000) demonstrated that running-induced lower limb muscle fatigue increases the tibia’s vertical acceleration during heel strike, suggesting that more load accumulates in the shank after running fatigued. Research by Mastalerz (2021) reported that the asymmetry of muscle activity on bilateral limbs was increased with the occurrence of running fatigue, suggesting that bilateral lower extremity muscles have different fatigue resistance. Typically, fatigue in the lower limb muscles reduces the cushioning effect of the muscles on the joint load. Therefore, the joint load tends to focus on one limb during running-induced fatigue, and the risk of unilateral lower limb injury increases (Furlong and Egginton, 2018). Radzak and colleagues (Radzak et al., 2017) found an increase in knee stiffness and internal rotation angle asymmetry after performing a running-induced fatigue test. In addition, a recent systematic review of the relationship between fatigue and symmetry shows that the effect of running fatigue on bilateral lower limb symmetry is not apparent, and the reasons may be related to experimental design, quantitative indicators, and test protocol (Heil et al., 2020). Running-induced fatigue may cause subtle or worsening existing gait asymmetry (Gao et al., 2020a). The assessment of bilateral limb asymmetry in functional movement is an essential measure of injury prediction and screening (Kiesel et al., 2007). In addition, a recent study demonstrates that reduced asymmetry in vertical impact peaks was associated with running injuries (Ceyssens et al., 2019). Therefore, a further factor that contributes to damage during long-distant running tasks is the presence of asymmetry (Gao et al., 2022b). It is particularly important to properly evaluate the effects exercise-induced fatigue on interlimb asymmetry to understand the underlying mechanisms of non-contact injury and improve exercise efficiency (Smeets et al., 2019).

The evaluation approaches of running gait symmetry have been widely utilized in biomechanical studies in past decades (Zifchock et al., 2006; Nigg et al., 2013; Radzak et al., 2017). Typically, the traditional classification theory of symmetry, such as symmetry index (SI) (Herzog et al., 1989) and symmetry angle (SA), was based on discrete variables like the mean peak GRF (Viteckova et al., 2018). However, the time-series information may be lost in the evaluation process of continuous data, such as a complete period of gait stance (Tabor et al., 2021). Therefore, symmetry function (SF), a novel symmetry checking method, was proposed by Nigg to evaluate continuous data with time dimensions (Nigg et al., 2013). Moreover, linear principal component analysis (PCA) has been used in the previous study to extract the gait characteristics of bilateral lower limbs and examine the degree of gait symmetry (Sadeghi, 2003). However, the limitation of SF and PCA is that only linear features of gait can be extracted, which reduces the model’s sensitivity in evaluating the non-linear features of time series parameters (Chau, 2001). The previous study demonstrated that the support vector machine (SVM) has a high generalization ability for dichotomous data (Figueiredo et al., 2018). The optimal separating hyperplane was created by maximizes the distance of separation in SVM model (Figueiredo et al., 2018). Moreover, it can transform the matrix into a higher dimensional space for classification by setting the different types of kernel functions (Figueiredo et al., 2018; Zhang et al., 2023). SVM algorithms was widely used in gait patterns recognition, such as differences of young and elderly populations (Eskofier et al., 2013), competitive and recreational runners (Clermont et al., 2017), barefoot and shod populations (Gao et al., 2022a). Given the gait differences caused by running fatigue, there is still a lack of understanding of the gait characteristics of dominant and non-dominant limbs and how these differences are attributed to limb injury prevention and function performance. Furthermore, the automatic detection of asymmetric gait and fatigue gait was lacking in the previous studies. Consequently, the SVM based on the structural risk minimization principle, were adopted to evaluate bilateral lower limb non-linear differences in this study (Chau, 2001).

This study aims to examine the asymmetry changes of bilateral gait characteristics before and after running fatigue and to develop a SVM machine learning model for asymmetry and fatigue gait recognition. It was hypothesized that the GRFs on both sides are asymmetrical, increasing with fatigue intervention. The second hypothesis is that non-linear kernels perform better than linear kernels for SVM models that recognize asymmetric and fatigue gaits.

## 2 Materials and methods

### 2.1 Participants

This study recruited fourteen male amateur runners who ran at least twice a week for less than 45 min or less than 10 km. The demographic information is given in Table 1. All subjects were asked to complete a questionnaire to determine whether the right limb was the dominant limb (the ball kicking leg) and had not suffered any lower limb or pelvic injuries in the previous 6 months. In addition, individuals with a history of lower extremity surgery were also excluded. The subjects have agreed in writing all the experiment contents, and the Ethics Committee of Ningbo University has approved this test protocol.

**TABLE 1:** Descriptive characteristics of 14 participants.

Information	Mean	SD
Age (year)	22.93	1.07
Height (cm)	176.83	2.61
Weight (kg)	70.17	6.57
BMI (kg/m^2^)	23.19	0.96

### 2.2 Data collection

**FIGURE 1:**
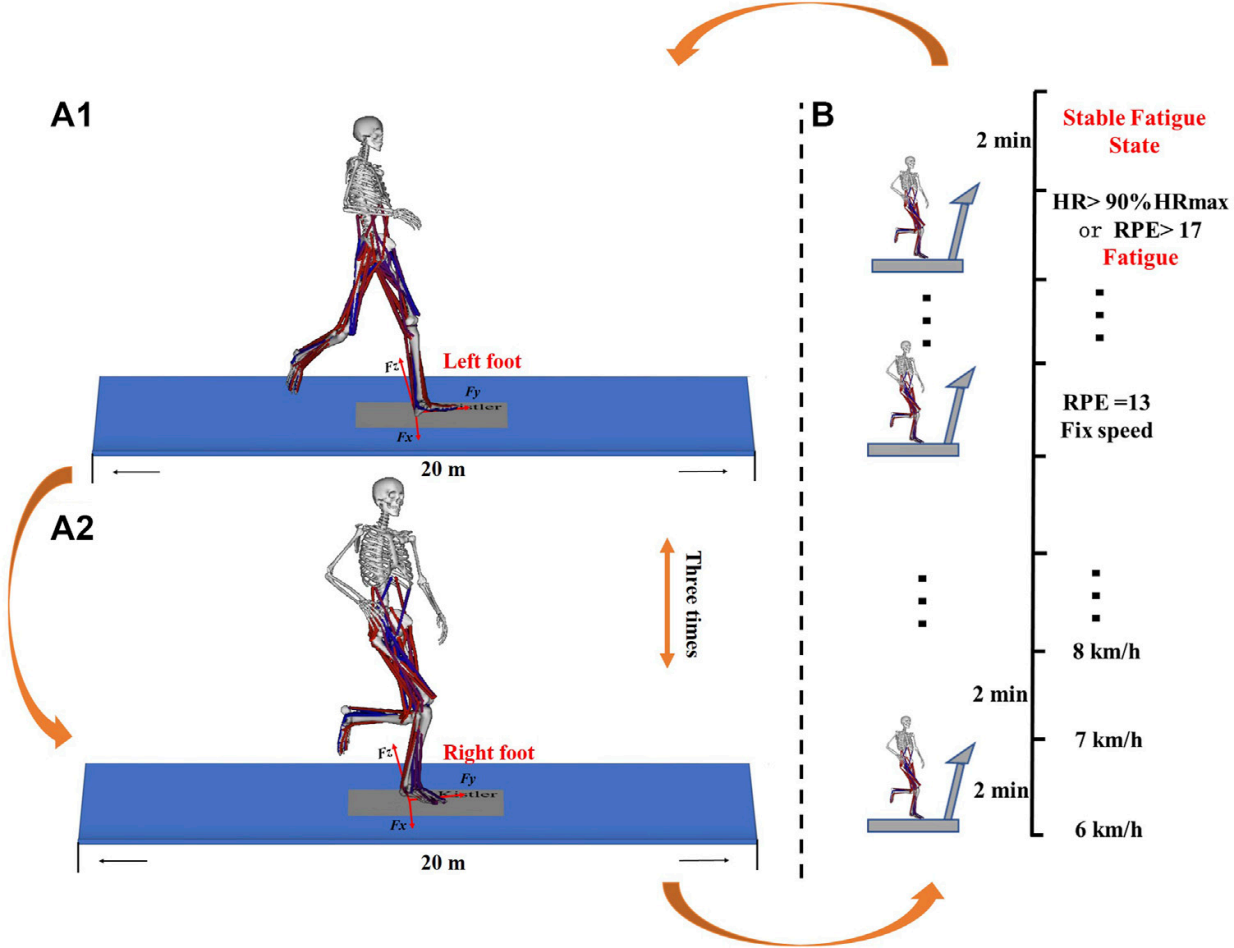
Bilateral data collection execution process. **(A1)** Right GRF data collection process. **(A2)** Left GRF data collection process. **(B)** Running-induced Fatigue Protocol Test implementation process.

Before this experiment, subjects were fully familiarized with the environment and processed a 10-min treadmill warm-up. The complete experiment was divided into three parts: data collection of GRFs of bilateral lower limbs before running fatigue, running-induced fatigue experiment (on the treadmill), and collection of the GRFs of bilateral lower limbs after running fatigue, as shown in Figure 1. The same acquisition process was applied with two GRF data collection experiences (pre- and post-fatigued).

As shown in Figure 1A1, a 3-dimensions force measuring plate (Sampling rate: 1,000 Hz) was embedded on a 20-m runway (Kistler, Winterthur, Switzerland). The force measuring plate was calibrated before each subject performed the test task. The participants determine the optimal distance from the force plate by familiarizing runway to increase the likelihood of effective contact between bilateral feet and the force plate during the runway trials. Whereafter, participants were instructed to run across a runway at a comfortable speed after familiarization and practices on the runway. During testing, the right foot didn’t land in the middle of the plate and the participants has active aiming force plate behavior were excluded (Figure 1A1). After a successful running trial has been performed, the same running mode was replaced with the left foot. until the three successful GRF data of the left and right sides were respectively collected (Figure 1A2). The second step was to implement the Running-induced Fatigue Protocol Test developed by Koblbauer and their colleagues (Koblbauer et al., 2014). The specific execution procedures have been reported in the previous study (Koblbauer et al., 2014). As shown in Figure 1A2, Heart rate (Polar RS100, Polar Electro Oy, Woodbury, NY, USA) and RPE (Ratings of Perceived Exertion, RPE, 6–20 Borg’s scale) were collected every 2 min throughout the procedure. The running speed increases by 1 km/h every 2 min until RPE = 13. Continue running at a fixed speed for 2 min after fatigue (HR>90%HRmax or RPE>17, HRmax = 200-age) (Gao et al., 2020a). The GRF data collection process was repeated until three successful data were collected on the left and right sides after the running-induced fatigue protocol test (Figure 1A1).

### 2.3 Data processing

GRF time series data of one support period were determined by vertical GRF = 30 N as the heel initial contact and toe-off phases (Quan et al., 2021). The GRF data of the time series were normalized to 101 frames by Cubic B-spline interpolation or extrapolation function in MATLAB (Version: R2019a, The MathWorks, Natick, MA, United States). All GRF data were standardized by individual body weight (GRF (BW) is equal to GRF divided by 10 times the body weight of the individual).

SF developed by Nigg and colleagues (Nigg et al., 2013) has high robustness for quantifying the symmetry of time series data, which can effectively avoid the data loss caused by the symmetry evaluation of discrete data. The symmetry time-varying information of 101-time points in SF can reflect the supporting stage of gait (Eq. 1). The degree of asymmetry is positively correlated with the value of SF, where the closer SF is to 0, the more symmetric the parameters are (Eq. 2). ±5% was set as an asymmetric threshold in the evaluation of this study (Winiarski et al., 2021).

$$SF=\int_{t=t_{1}}^{t_{2}}\;A\left|x_{r}\left(t\right)-x_{l}\left(t\right)\right|dt$$ (1)

$$A=\frac{2}{range\left(x_{r}\left(t\right)\right)+range\left(x_{l}\left(t\right)\right)}$$ (2)

Where $x_{r}\left(t\right)$ and $x_{l}\left(t\right)$ were defined as the value of the GRF for the right and left foot at the time t, respectively. The $t_{1}$ is the time at heel strike point during stance phase and $t_{2}$ is the time at take-off point during the stance phase.

### 2.4 Machine learning model building

SVM is a machine learning algorithm that realizes data dichotomy through case learning (Teufl et al., 2019). It has been widely used in biology and medicine (Noble, 2006). Such as the automatic classification of DNA sequences, gene expression profiles from tumor samples or peripheral fluids, microarray expression profiles, and mass spectrometry (Golub et al., 1999). Compared with other machine learning algorithms, such as neural networks, the SVM algorithm based on supervised learning has better robustness and generalization for gait symmetry analysis (Wu and Wu, 2015). In this study, six SVM models were constructed to evaluate the characteristic differences of GRF in the medial-lateral (X), anterior-posterior (Y) and vertical (Z) directions of left and right feet and before and after fatigue. Among them, the left GRF and SF of Pre-fatigue belong to feature 1 (X, Y, and Z directions, respectively). The right GRF and SF of Post-fatigued belong to feature 2 (X, Y, and Z directions, respectively). Optimal symmetric gait and fatigue gait recognition model by adjusting the non-linear kernel function. The kernel function $K\left(x_{i},x\right)=\phi \left(x_{i}\right)\phi \left(x\right)$ based on Mercer theorem (Vapnik, 1999).

The difference of non-linear gait features between dominant and non-dominant limbs was identified by the SVM algorithm in MATLAB (Version: R2019a, The MathWorks, Natick, MA, United States). The first step is to map the non-linear function ϕ : $R^{b}\to F$ to high dimensional feature space W. In addition, the optimal linear classification plane f in W was determined by feedback learning. The training set data sample D is assumed by Eq. 3.

$$D={\left\{\left(x_{i},y_{i}\right)\right\}}_{i=1}^{n}$$ (3)

Where each raw output quantity is defined as $x_{i}\in R^{b}$ B and n represent the original input data’s feature dimension and sample size, respectively.

$$f:R^{b}\to \left\{-1,+1\right\}$$ (4)

$$x_{i}\to y_{i}$$ (5)

In the training set data, two gait pattern data are mapped to the corresponding class flag space $y_{i}\in \left\{-1,+1\right\}$ , − 1 in each SVM model in this study, the parameters in features 1 and + 1 are the parameters in feature 2. In addition, the optimal linear classification surface f in the model is obtained by Eq. 6

$$f\left(x\right)=sgn\left(\overset{n}{\underset{i=1}{\sum }}\beta_{i}y_{i}K\left(x_{i},x\right)+b\right)$$ (6)

$$minW\left(\beta \right)=-\beta^{T}I+\frac{1}{2}\beta^{T}D\beta$$ (7)

$$s.t.\;\beta^{T}y=0;\beta_{i}\in \left[0,C\right]$$ (8)

where *b* and $\beta_{i}$ are defined as estimation bias and optimal classification hyperplane coefficients for training set data, respectively. *C* is the penalty factor, which determines the distance between the support vector and the decision plane. Parameter G is mainly used for height mapping of low-dimensional samples, and the larger the value of g, the higher the dimension of the mapping. Optimal classification hyperplane coefficients can be obtained by solving quadratic programming problems in Eqs 7, 8. In the SVM model with stable inseparable linear data, the balance between the maximum interval and the minimum training error is adjusted by setting the best *C* and best G parameter.

In this study, the accuracy of the SVM model was evaluated by the five-fold cross-validation method (Begg and Kamruzzaman, 2005). The optimal machine learning model was determined based on different kernel function types and parameters by adjustment. The following three commonly used kernel function types were selected to evaluate the generalization ability of the SVM model in this study.

Linear kernel function (LINEAR) is shown in Eq. 9:

$$K\left(x_{i},x\right)=x_{i}x$$ (9)

Gaussian radial basis kernel function (RBF) is shown in Eq. 10:

$$K\left(x_{i},x\right)\exp\left(-\frac{{∥x_{i}-x∥}^{2}}{2\sigma^{2}}\right)$$ (10)

The polynomial kernel function (POLY) is shown in Eq. 11:

$$K\left(x_{i},x\right)={\left(x_{i}x+1\right)}^{d}$$ (11)

where the *d* is defined as the order of the POLY.

Classification Accuracy (ACC), Sensitivity (SEN), and Specificity percentage (SEP) are used to evaluate the performance of classification machine learning models (Begg and Kamruzzaman, 2005). Thereinto, *Ac* (12) was used to evaluate the model’s ability to strive for recognition of the two features. *Se* (13) and *Sp* (14) were used to evaluate the model’s ability to correctly identify the first feature and the second feature, respectively.

$$ACC=\frac{T_{1}+T_{2}}{T_{1}+F_{1}+T_{2}+F_{2}}\times 100\%$$ (12)

$$SEN=\frac{T_{1}}{T_{1}+F_{2}}\times 100\%$$ (13)

$$SEP=\frac{T_{2}}{T_{2}+F_{1}}\times 100\%$$ (14)

where, T1 and T2 are the correct recognition formats of feature 1 and feature 2 by the SVM model, and F1 and F2 are the number of feature 1 and feature 2 incorrectly recognized by the SVM model, respectively.

### 2.5 Statistical analysis

In this study, The Shapiro-Wilks test was used to verify the normality of the data prior to statistical analysis. SF was used to evaluate the symmetry degree of bilateral GRF, and the paired sample *T*-test in the statistical Parameter Mapping (SPM) algorithm was used as the different test of bilateral gait characteristics. In addition, the paired sample *T*-test in SPM was also used to check SF of GRF changes in X, Y, and Z directions before and after fatigue in MATLAB (Version: R2019a, The MathWorks, Natick, MA, United States). The significance level was set to 0.05.

## 3 Results

### 3.1 Pre-fatigue biomechanical variable

**FIGURE 2:**
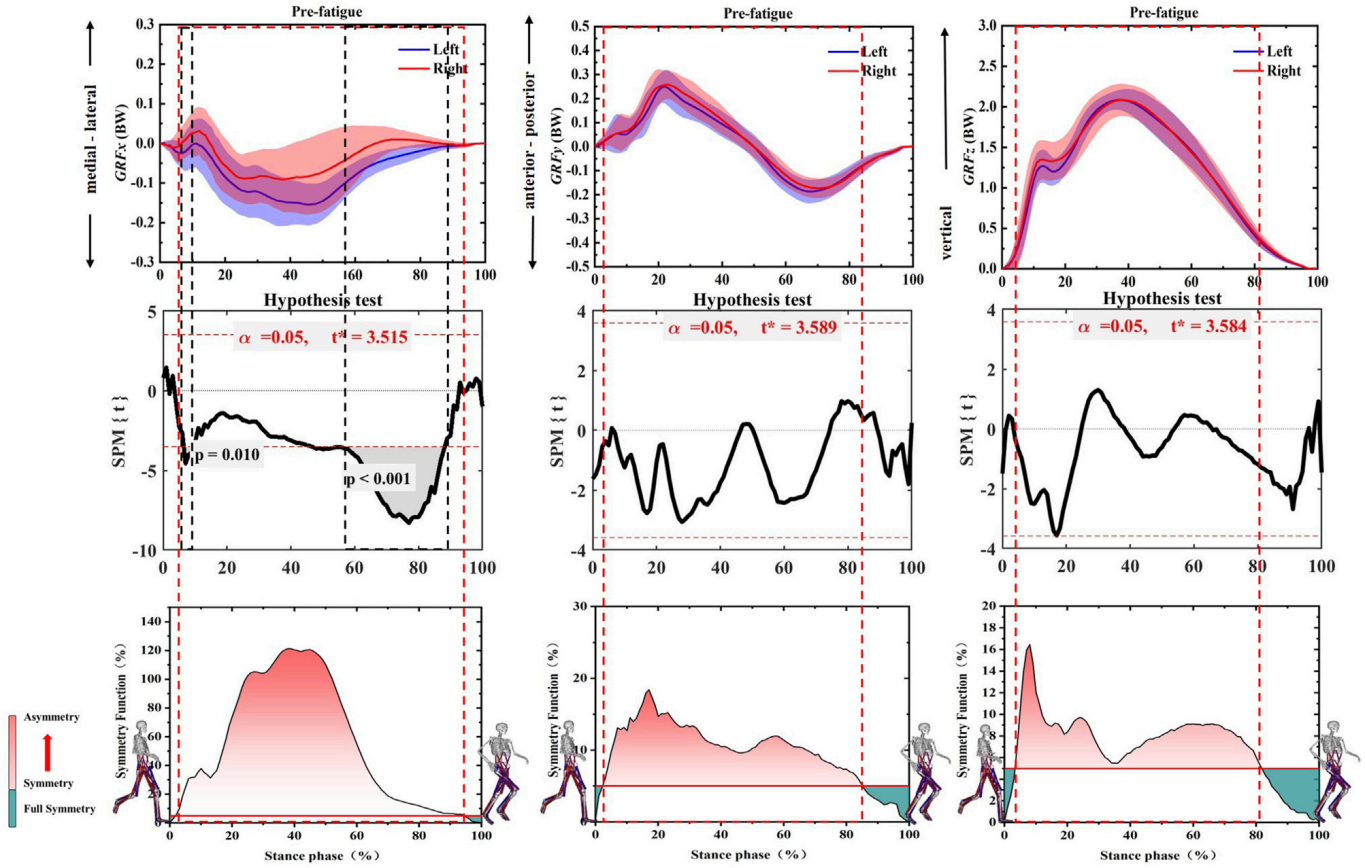
Illustration of SF degree and SPM test of GRFx, GRFy, and GRFz during the whole gait stance phase before Running-induced Fatigue Protocol Test. Note: The red dotted boxes represent asymmetrical degrees. The black dotted boxes represent a significant difference (*p* < 0.05). The red fill represents the degree of asymmetry, and the darker the color, the more asymmetry. The green fill represents full symmetry. GRFx: medial-lateral direction of GRF, GRFy: anterior-posterior direction of GRF, GRFz: vertical direction of GRF.

There are asymmetries in all three directions before Running-induced Fatigue Protocol Test, as shown in Figure 2. Specifically, the SF of GRFx rise steadily until the peak value of 1.2 at 3%–40% of the gait stance phase, and then it declines to 0.05 at 95% of the gait stance phase. In addition, the SPM results showed that the non-dominant limb appears to have greater medial direction force on 7%–10% (*p* = 0.010, t = 3.515) and 58%–90% of the gait stance phase (*p* < 0.001, t = 3.515). In addition, the SF of GRFy and GRFz were more than 0.05 in 3%–85% and 4%–80% of the gait stance phase, respectively.

### 3.2 Post-fatigued biomechanical variables

**FIGURE 3:**
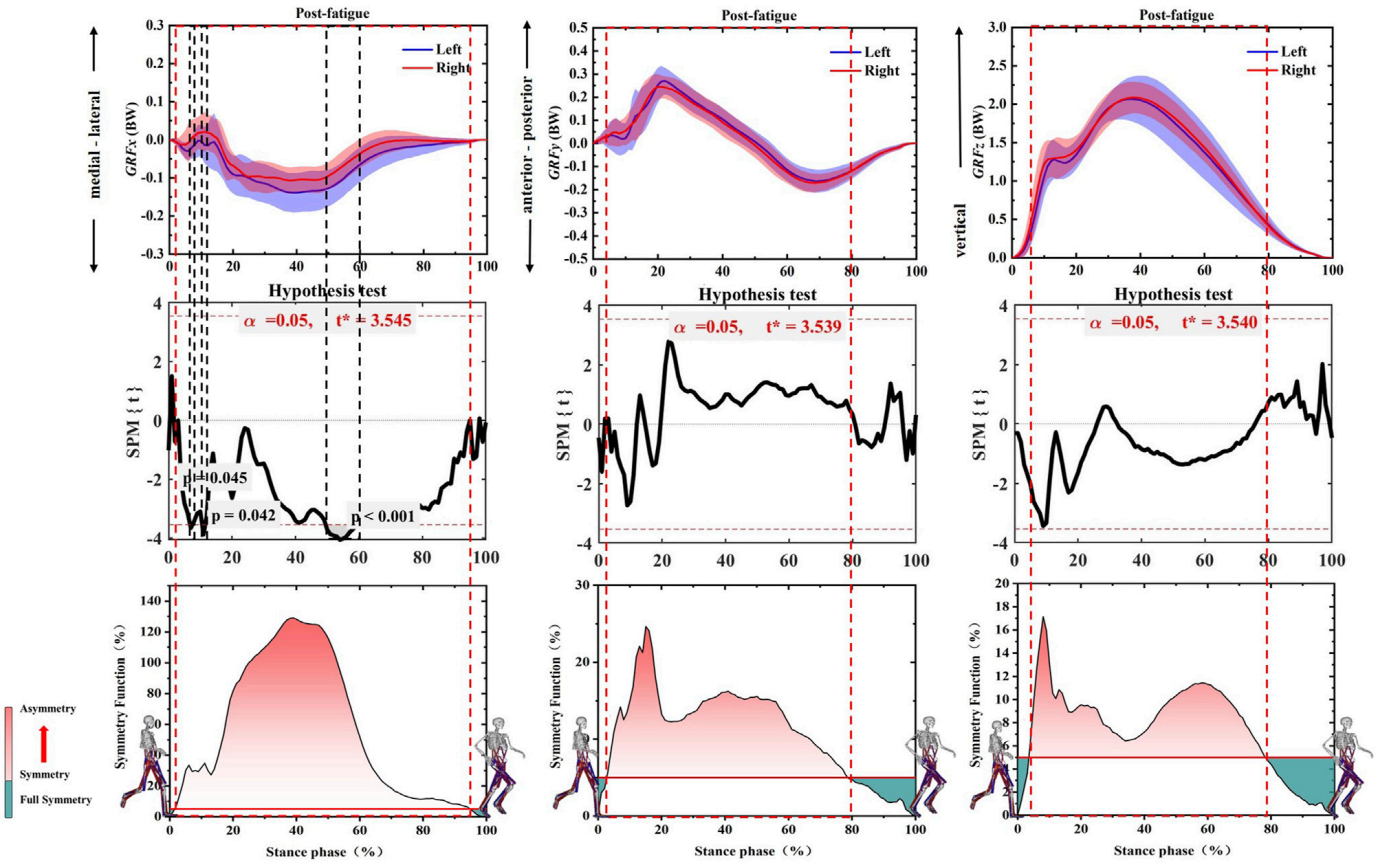
Illustration of SF degree and SPM test of GRFx, GRFy, and GRFz during the whole gait stance phase after Running-induced Fatigue Protocol Test. Note: The red dotted boxes represent asymmetrical intervals. The black dotted boxes represent a Significant difference. The red fill represents the degree of asymmetry, and the darker the color, the more asymmetry. The green fill represents full symmetry. GRFx: medial-lateral direction of GRF, GRFy: anterior-posterior direction of GRF, GRFz: vertical direction of GRF.

As shown in Figure 3, asymmetry of GRFx, GRFy, and GRFz was observed on both sides after the Running-induced Fatigue Protocol Test. The SF of GRFx exceeded 1.0 in 30%–55% of the gait stance phase. the SPM results showed that the left GRFx exhibited greater medial force on 8%–9% (*p* = 0.045, *t* = 3.545), 10%–12% (*p* = 0.042, *t* = 3.545), and 50%–60% (*p* < 0.001, *t* = 3.545) of the gait stance phase. Moreover, the SF of GRFy and GRFz were more than 0.05 in 3%–80% and 5%–79% of the gait stance phase, respectively.

### 3.3 Symmetry function of pre- and post-fatigue

**FIGURE 4:**
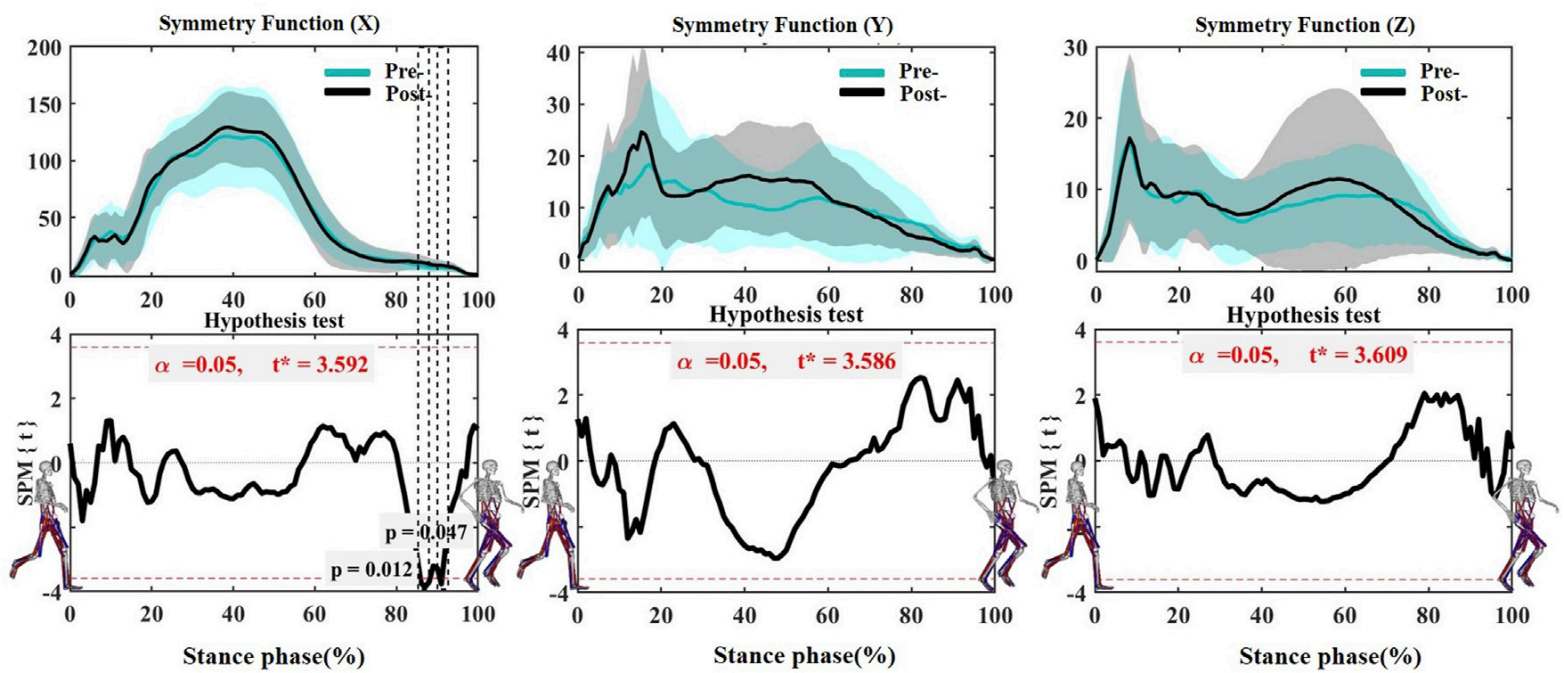
Comparing the mean values of SF of GRFx, GRFy, and GRFz from all participants between Pre-fatigue and Post-fatigue. Note: Pre-is Pre-fatigue, and Post-is Post-fatigue. The black dotted boxes represent a significant difference (*p* < 0.05). GRFx: medial-lateral direction of GRF, GRFy: anterior-posterior direction of GRF, GRFz: vertical direction of GRF.

According to the SPM inspection results (Figure 4), Only SF of GRFx changed significantly after the Running-induced Fatigue Protocol Test. SF of post-fatigued was significantly higher than that pre-fatigue at 86%–88% (*p* = 0.012, *t* = 3.592) and 90%–92% (*p* = 0.047, *t* = 3.592) of the gait stance phase. GRFy and GRFz did not change significantly after the Running-induced Fatigue Protocol Test.

### 3.4 SVM model generalization ability

#### 3.4.1 Left and right SVM models

**FIGURE 5:**
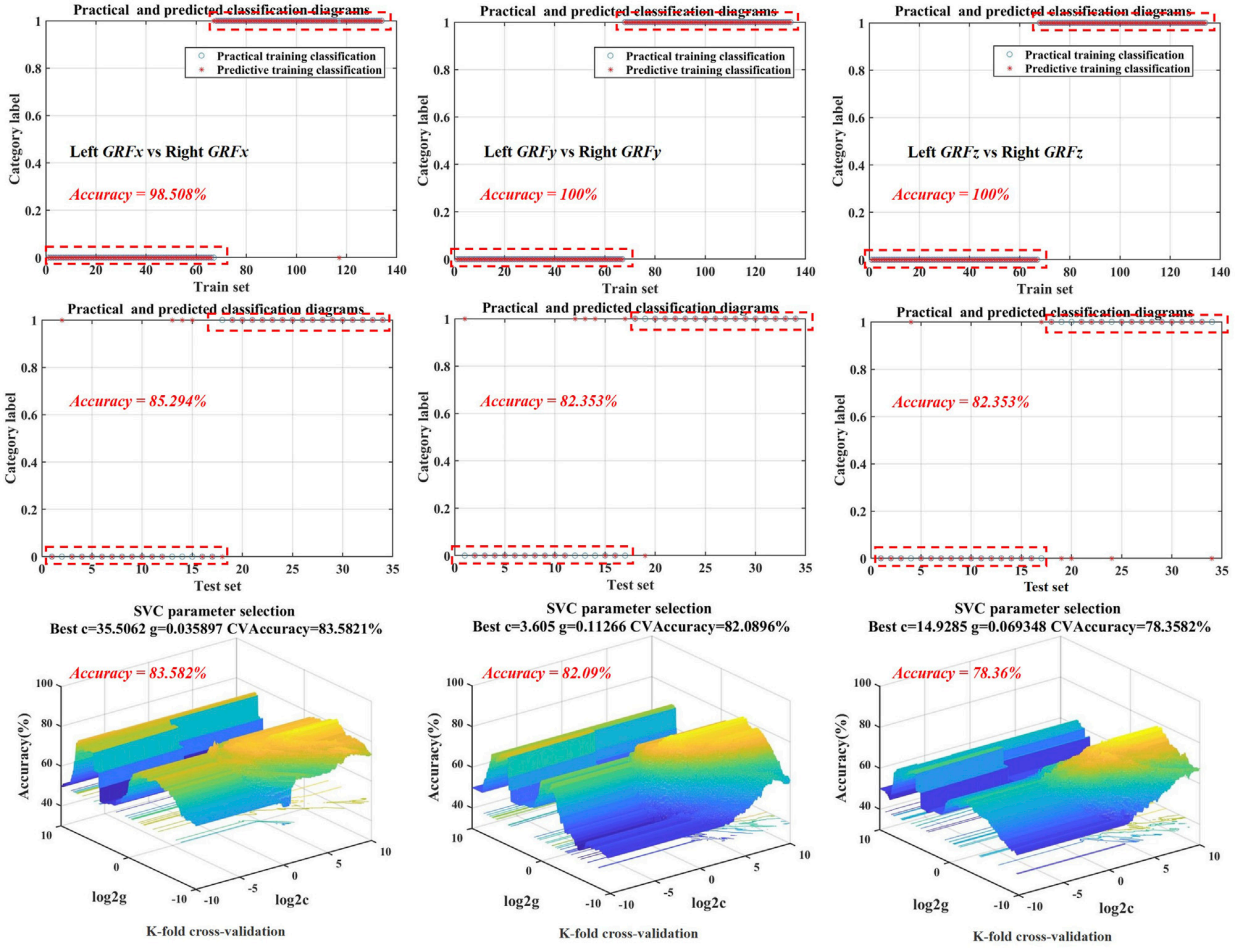
The training set, test set, and cross-validation model accuracy visualization are based on optimal kernel function selection for SVM models of GRFx, GRFy, and GRFz. GRFx: medial-lateral direction of GRF, GRFy: anterior-posterior direction of GRF, GRFz: vertical direction of GRF.

It can be seen from Table 2 that the optimal kernel functions of SVM models of GRFx, GRFy, and GRFz are all POLY. ACC, SEN, and SEP of the SVM model of GRFx were achieved at 85.3%, 84.2%, and 93.3%, respectively. Through model cross-validation, best C, Best G, and CVAcc are 33.506, 0.036, and 0.836, respectively. The prediction accuracy of the test set of GRFx reached 85.294%, as shown in Table 2 and Figure 5. In addition, ACC, SEN, and SEP of GRFy SVM model were achieved at 82.4%, 76.2%, and 92.3%, respectively. The prediction accuracy of the test set of GRFy reached 82.35%, as shown in Figure 4. Through model cross-validation of the SVM model of GRFy, the Best C, Best G, and CVAcc are 3.605, 0.113, and 0.821, respectively. Moreover, the SVM Model of GRFz showed 82.35% prediction accuracy for the test set (Figure 4). ACC, SEN, and SEP of the SVM model of GRFz were achieved at 82.4%, 86.7%, and 78.9%, respectively, and the Best C, Best G, and CVAcc are 14.929, 0.069, and 0.784, respectively.

**TABLE 2:** Comparison of results from the different SVM classification algorithms designed for SVM models of GRFx, GRFy, and GRFz. Note: The best kernel type is highlighted in bold. GRFx: medial-lateral direction of GRF, GRFy: anterior-posterior direction of GRF, GRFz: vertical direction of GRF.

	Kernel	Cross-validation results			Model precision		
		Best C	Best G	CVAcc	ACC	SEN	SEP
	LINEAR	35.506	0.036	0.836	0.794	0.857	0.75
**GRF*x***	RBF	35.506	0.036	0.836	0.824	0.762	0.923
	**POLY**	35.506	0.036	0.836	**0.853**	**0.842**	**0.933**
	LINEAR	3.605	0.113	0.821	0.794	0.813	0.778
**GRF*y***	RBF	3.605	0.113	0.821	0.676	0.667	0.688
	**POLY**	3.605	0.113	0.821	**0.824**	**0.762**	**0.923**
	LINEAR	14.929	0.069	0.784	0.677	0.75	0.652
**GRF*z***	RBF	14.929	0.069	0.784	0.824	0.824	0.824
	**POLY**	14.929	0.069	0.784	**0.824**	**0.867**	**0.789**

#### 3.4.2 SF of pre-fatigue and post-fatigued SVM models

It can be seen from Table 3 that the optimal kernel functions of SVM models of SFx, SFz are POLY, and the optimal kernel function of SFy is POLY. ACC, SEN, and SEP of the SVM model of SFx were achieved at 62.5%, 66.7%, and 60%, respectively. Through model cross-validation, Best C, Best G, and CVAcc are 1.569, 0.043, and 0.75, respectively. The prediction accuracy of test set reached 62.5%, as shown in Table 3 and Figure 5. In addition, ACC, SEN, and SEP SVM models of SFy were achieved 54.2%, 100%, and 52.2%, respectively. In addition, the prediction accuracy of the test set reached 54.167%, as shown in Figure 5. Through model cross-validation of the SVM model of SFy, the Best C, Best G, and CVAcc are 20.393, 0.002, and 0.633, respectively. Moreover, the SVM model of SFz showed 62.5% prediction accuracy of the test set (Figure 4). ACC, SEN, and SEP of SVM model of SFz were achieved 62.5%, 74%, and 53%, respectively, and the Best C, Best G, and CVAcc are 0.001, 9.514, and 0.617, respectively.

**TABLE 3:** Comparison of results from the different SVM classification algorithms designed for SVM models of SFx, SFy, and SFz before and after the Running-induced Fatigue Protocol Test. Note: The best kernel type is highlighted in bold, SFx: medial-lateral direction of SF, SFy: anterior-posterior direction of SF, SFz: vertical direction of SF.

	Kernel	Cross-validation results			Model precision		
		Best C	Best G	CVAcc	ACC	SEN	SEP
	LINEAR	1.569	0.043	0.75	0.5	0.5	0.5
**SF*x***	**RBF**	1.569	0.043	0.75	**0.625**	**0.667**	**0.6**
	POLY	1.569	0.043	0.75	0.625	0.636	0.615
	LINEAR	20.393	0.002	0.633	0.542	0.556	0.533
**SF*y***	RBF	20.393	0.002	0.633	0.56	0.6	0.533
	**POLY**	20.393	0.002	0.633	**0.542**	**1**	**0.522**
	LINEAR	0.001	9.514	0.617	0.5	0	0.5
**SFz**	**RBF**	0.001	9.514	0.617	**0.625**	**0.75**	**0.63**
	POLY	0.001	9.514	0.617	0.5	0.5	0.5

## 4 Discussion

This study aimed to examine the effect of running fatigue on bilateral GRF symmetry and develop SVM models to realize asymmetric gait and fatigue gait automated recognition. We found that GRF of bilateral lower limbs was asymmetric in all three directions (medial-lateral, anterior-posterior and vertical) at pre-fatigue states, especially a significantly worsened of asymmetry with the occurrence of running fatigue in the medial-lateral direction. In addition, the SVM model with POLY kernel has demonstrated to have higher accuracy for feature extraction of symmetric gait, while the SVM model with RBF kernel has higher accuracy for fatigue gait automated recognition in anterior-posterior and vertical directions. Furthermore, the POLY kernel had highlighted to have relatively higher accuracy for fatigue gait recognition in the vertical direction. In general, the results of this study are consistent with the previous hypotheses.

Gait asymmetry in healthy individuals may be related to the functional attributes of bilateral limbs (Sadeghi et al., 2000; Pan et al., 2023). Previous study have emphasized that the dominant limb usually plays a gait propulsion role while opposing limbs contribute to gait support and control (Sadeghi et al., 2000). The dominant limb during the gait cycle was associated with more power generation, which was mainly reflected in the positive anterior-posterior GRF impulse (Seeley et al., 2008). Therefore, the non-dominant limb is subjected to significant negative anterior-posterior GRF impulse (Potdevin et al., 2008). Interestingly, this study found statistical differences in medial-lateral direction of GRF during the push-off phase before the fatigue intervention, suggesting the neuromuscular control asymmetry in healthy individuals (Radzak et al., 2017). The non-dominant limb maintains the stability of the gait stance phase through the greater medial force (Sadeghi et al., 2000). Similarly, the larger SF of medial-lateral direction of GRF present in the stance phase of the entire stance phase can also explain this idea. The only slight asymmetry of vertical direction of GRF (SF < 18) and no significant difference before and after fatigue were reported in the current study, which contradicted the previous reports that vertical direction of GRF was a major variable of symmetry in running gait (Gao et al., 2020b).

**FIGURE 6:**
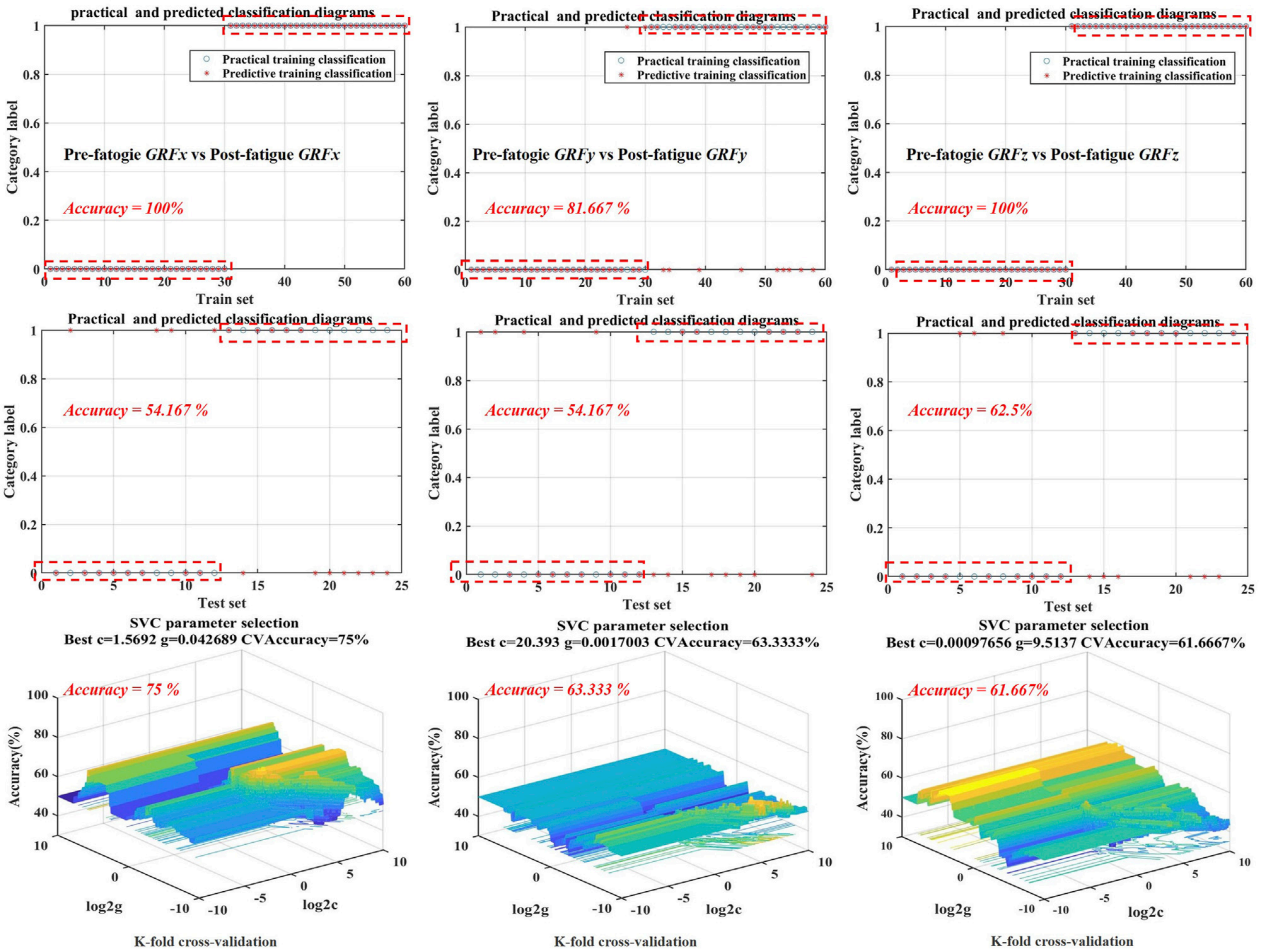
Training set, test set and cross-validation model accuracy visualization are based on optimal kernel function selection for SVM models of SF for SVM models of SF*x*, SF*y* and SF*z* before and after Running-induced Fatigue Protocol Test. SFx: medial-lateral direction of SF, SFy: anterior-posterior direction of SF, SFz: vertical direction of SF.

The kinematics and kinetics variables of bilateral lower limbs may be changed due to the weakened central nervous control over muscles during long-distance running (Quan et al., 2021). Our findings are in line with a previous argument that gait asymmetry increases with fatigue (Gao et al., 2020a). The current study report that the asymmetry was mainly found in the medial-lateral direction, and it was observed to occur in the heel contact stage (8%–9% and 10%–12%) and the mid-foot forward transition stage (50%–60%), suggesting that the more significant medial load exist in the non-dominant foot during running gait after fatigue intervention. This phenomenon may be the potential cause of ankle pronation and arch collapse after long-distance running (Fourchet et al., 2015). Moreover, The asymmetry of anterior-posterior and vertical direction of GRF was observed to occur before the push-off period, which was consistent with the performance before fatigue, suggesting that fatigue did not affect the asymmetry of GRF in anterior-posterior and vertical directions (Van Gheluwe and Madsen, 1997). Moreover, it can be seen from Figure 6 that the symmetry of medial-lateral direction of GRF deteriorates during the push-off phase of gait (86%–88% and 90%–92%) after fatigue intervention, suggesting that the more medial load was concentrated in the metatarsal joint of non-dominant foot (Gao et al., 2020a). The deterioration of this asymmetry may be related to the risk of overuse injury to the unilateral metatarsal-toe joint, possibly due to a higher susceptibility to fatigue in the lower limb muscles of the non-dominant limb, but further research is needed to verify if GRF measures could be used to infer foot neuromuscular control.

Sensitivity and specificity were used to measure the ability of the classifier to detect gait patterns of left and right limbs and before and after fatigue, respectively (Chan et al., 2002). Earlier, Chan et al. (Chan et al., 2002) reported 91% and 94% similarity success rates for second-order POLY and linear nuclei in an SVM gender classification task based on gait video sequence data. Our results also show that the SVM model can map the underlying data structures related to asymmetrical and fatigue gait (Atanassov et al., 2021). Machine learning-based classifiers can automatically recognize particular gait patterns according to their measurement methods, which is expected to provide a basis for exploring the potential biomechanical mechanism of running-fatigued. Early recognition of gait problems caused by symmetry changes by a machine classifier can avoid fatigue through a motor intervention program in advance, thus reducing the incidence of injuries caused by fatigue and asymmetry gait. Compared with traditional symmetry evaluation methods, the SVM algorithm based on non-linear kernel function mapping data to high-dimensional space classification has a more substantial symmetry quantization ability (Wu and Wu, 2015).

In this study, it is observed that the adjustment of optimal parameters is also crucial to the improvement of model accuracy, especially the choice of penalty parameter C and kernel function. The change of each kernel will lead to a change in the model. Therefore, the optimal model is obtained through repeated testing of a large number of experiments in this study (Begg and Kamruzzaman, 2005). The selection of kernel function has an important influence on the generalization ability of the SVM model since the kernel function reflects the internal changes of gait biomechanical characteristics by mapping the mutual non-linear relationship between gait variables into the high-dimensional feature space (Schölkopf et al., 2002). Therefore, three kernel functions (RBF, POLY, and LINEAR) were selected for gait data analysis, considering that all gait parameters may have probability distributions in higher space. It can be seen from Tables 2, 3 that the generalization performance of non-linear kernel functions such as POLY and RBF is better than that of LINEAR in the SVM model of gait feature recognition. A recent study reported by Xiang and his colleagues (Xiang et al., 2022b) showed that the SVM model based on RBF had a prediction accuracy of 93% to classify dynamic plantar pressure and foot metrics of barefoot and shod people. This finding is consistent with the results obtained in this study that the prediction accuracy of SVM model is higher than that of the LINEAR. Specifically, the POLY kernel can make the SVM model the most accurate in classifying left and right gait features, with accuracy of 85.3%, 82.4%, and 82.4% in medial-lateral, anterior-posterior and vertical directions, respectively. Similarly, RBF was the optimal kernel function of the SVM model for fatigue gait recognition in medial-lateral (54.2%) and vertical direction (62.5%). Furthermore, POLY was the optimal kernel function of the anterior-posterior direction (54.2%). Using these SVM model to achieve automatic gait feature of bilateral lower limbs and of fatigue gait classification, suggesting that early recognition runners gait asymmetry and fatigue gait. As a final summary, SVM classifier within the healthy individuals of this study provides a basis for further exploring the automatic recognition methodology of gait asymmetry and of fatigue gait.

There are also four limitations to this study. Firstly, in the process of GRF data collection, we only used one force plate to measure the left and right gait characteristics, respectively. secondly, the participants in this study are all young groups, so the model may not be applicable to older runners. Future studies should consider the biomechanical data sets of all ages for model training. In addition, although the running-induced fatigue experiment is a classic fatigue method, it is performed on a treadmill and may differ from long-distance running on the ground, such as a marathon. Moreover, the only GRF data was considered as a variable for gait recognition in this study, more variables that can sensitively reflect asymmetric gait, such as joint Angle, should be included in the further study. At last, this study selected participants’ comfortable running speed for ground data collection, thus ignoring the possible effect of speed on GRF. Future studies should compare the GRF differences between comfortable and standard running speeds.

## 5 Conclusion

In this study, the asymmetry degree and changes of GRF in both lower limbs of 14 amateur male runners were investigated before and after a running-induced fatigue experiment. An SVM machine learning model was established to mine and recognize the characteristics of left and right gait and fatigue gait non-linear. The findings of this study suggest that GRF asymmetry existed in the medial-lateral, anterior-posterior and vertical directions of bilateral lower limbs, especially in medial-lateral direction. In addition, the asymmetry of GRF in the medial-lateral direction was increased after fatigued. Moreover, the POLY and RBF kernel contribute more to recognizing asymmetric and fatigue gait characteristics in SVM machine learning model, respectively.

## Data Availability

The raw data supporting the conclusion of this article will be made available by the authors, without undue reservation.
